# Spindle epithelial tumor with thymus‐like element (SETTLE)—Report of a rare thyroid carcinoma

**DOI:** 10.1002/ccr3.9300

**Published:** 2024-08-10

**Authors:** Patrícia Bernardo, Henrique Messias, Ricardo Nogueira, Brazão Lopes, Pedro Gomes

**Affiliations:** ^1^ General Surgery Department ULS Arco Ribeirinho Barreiro Portugal; ^2^ Head and Neck Surgery Department Instituto Português de Oncologia de Lisboa Francisco Gentil Lisbon Portugal; ^3^ Division of Clinical and Surgical Sciences University of Edinburgh Edinburgh UK; ^4^ Pathological Anatomy Department Instituto Português de Oncologia de Lisboa Francisco Gentil Lisbon Portugal

**Keywords:** endocrinology and metabolic disorders, oncology, pathology and laboratory medicine, surgery

## Abstract

Spindle epithelial tumor with thymus‐like element should be included in the differential diagnosis of thyroid gland cancers, particularly in medullary carcinoma, younger patients and indolent clinical presentation, because it may influence treatment and prognosis.

## 1 INTRODUCTION

Spindle epithelial tumor with thymus‐like element (SETTLE) is a clinical entity first proposed by Chan and Rosai, in 1991.^1^ It is considered part of a group of cervical lesions sharing a common histogenesis related to thymic or branchial pouch remnants that also includes thymoma and carcinoma showing thymus‐like differentiation (CASTLE).^1^

SETTLE is a very rare thyroid carcinoma predominantly occurring in the early decades of life.^1^, ^2^ To date, no genetic or environmental predisposing factor has been found to be related to this neoplasm although single cases have been reported to show mutation of KRAS, NRAS, KMT3D, or KMT2C.^3^, ^4^ It is classified as a low‐grade malignancy usually presenting as a slow‐growing thyroid mass without distant metastasis.^5^ Also, patients are usually euthyroid and with normal serum calcitonin.^2^ Since it is very rare, no clinical guidelines have been established yet. Diagnosis can be challenging, requires high clinical suspicion and is confirmed by histopathological features.^6^ Surgery is the mainstay of therapy, with or without adjuvant treatment.^5^, ^7^ Despite being indolent, SETTLE may develop late metastasis, thus long‐ term follow‐up is recommended.^2^, ^5^, ^6^

The authors report a rare case of SETTLE of the thyroid gland, which was initially misdiagnosed as the more common medullary thyroid carcinoma. Additionally, the important clinical implications of the differential diagnosis are described, along with a review of current scientific literature.

## 2 CASE HISTORY/EXAMINATION

A 20‐year‐old female patient was referred to the Head and Neck surgery department with a history of a cervical mass that had been growing progressively for 10 months and associated with occasional shortness of breath with physical activity and a feeling of foreign body in the neck. The patient denied dysphonia, dysphagia, or other complaints. There was no relevant past medical or family history. Physical examination detected an anterior midline cervical mass that was firm and solid. Remaining head and neck and general physical examination was unremarkable.

## 3 METHODS (DIFFERENTIAL DIAGNOSIS, INVESTIGATIONS AND TREATMENT)

**FIGURE 1:**
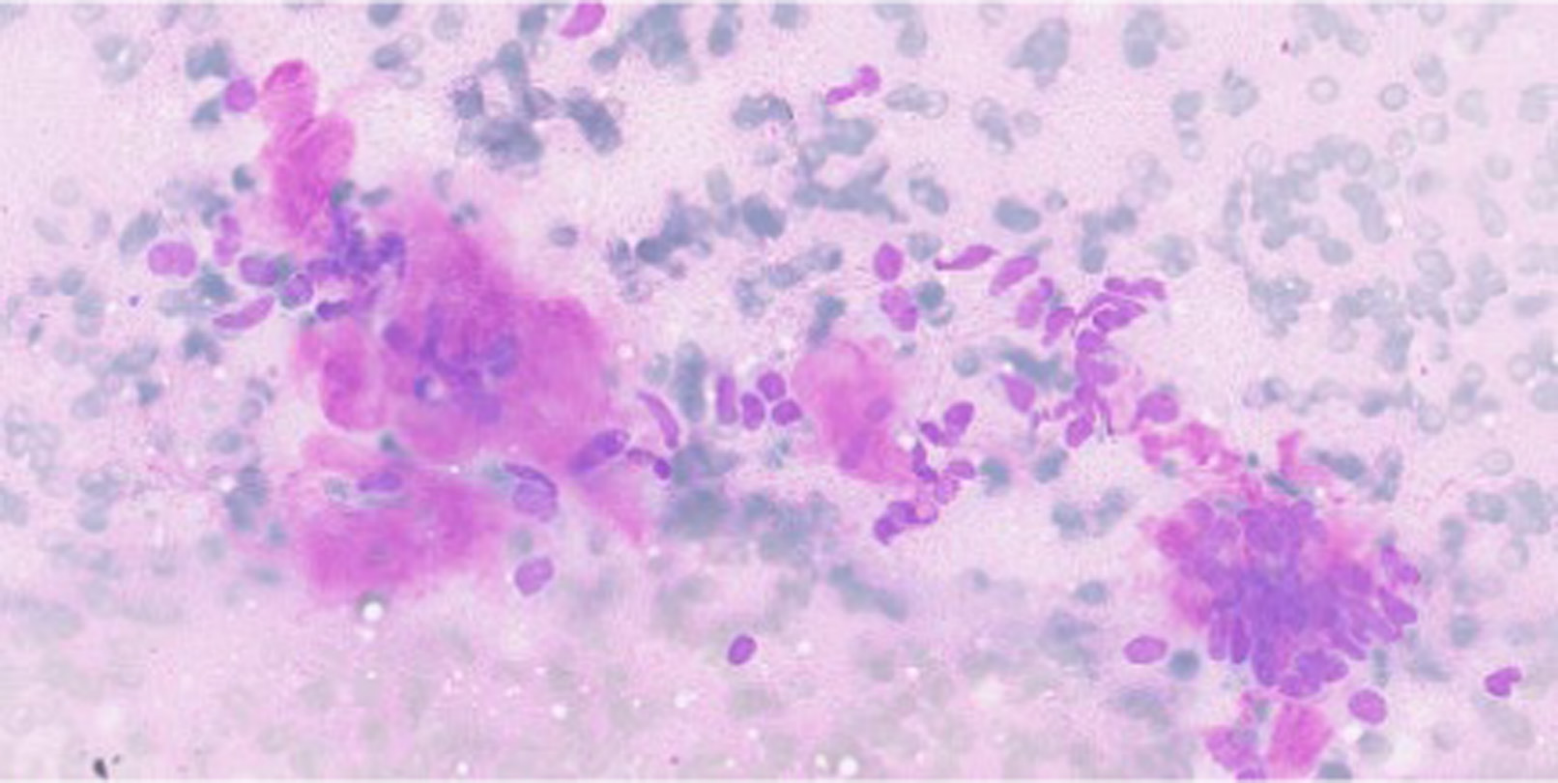
Fine‐needle aspirate smears showed groups of spindle cells in a background with amorphous metachromatic extracellular material, which was misinterpreted as amyloid (MGG × 400).

**FIGURE 2:**
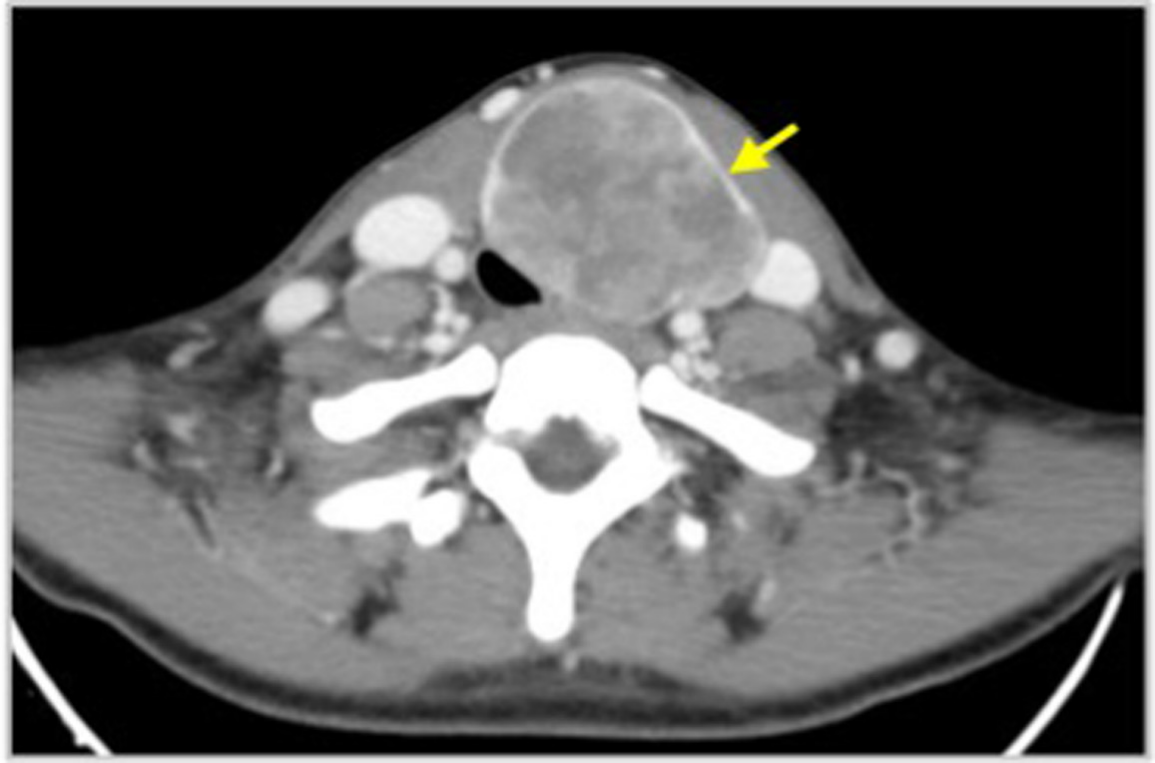
Computed axial tomography scan of the neck demonstrating the thyroid nodule filling almost the entire left lobe (arrow) with associated tracheal lumen reduction and tracheal right deviation.

All blood tests, including serum TSH, T4, thyroid antibodies, and calcitonin, were within normal value range. Ultrasonography of the neck identified a heterogeneous, hypoechoic solid nodule filling almost the entire left lobe, with 80 × 40 × 40 mm in size. No neck lymphadenopathies were detected. Fine‐needle aspiration cytology (FNAC) of the same nodule was suggestive of medullary carcinoma (Figure 1). Further diagnostic workup detected right deviation of the trachea, tracheal lumen reduction greater than 50% and erasing of peri‐esophageal fat on CT scan (Figure 2), but no signs of lumen invasion on bronchoscopy.

The case, which was initially suggestive of medullary thyroid carcinoma, was discussed in a thyroid oncology multidisciplinary team meeting and proposed for surgical treatment with total thyroidectomy and central compartment lymph node dissection. During surgery, however, it was decided not to perform the lymphadenectomy due to the absence of suspicious lymph nodes. The patient was discharged on the third postoperative day with no immediate complications.

## 4 CONCLUSION AND RESULTS (OUTCOME AND FOLLOW‐UP)

**FIGURE 3:**
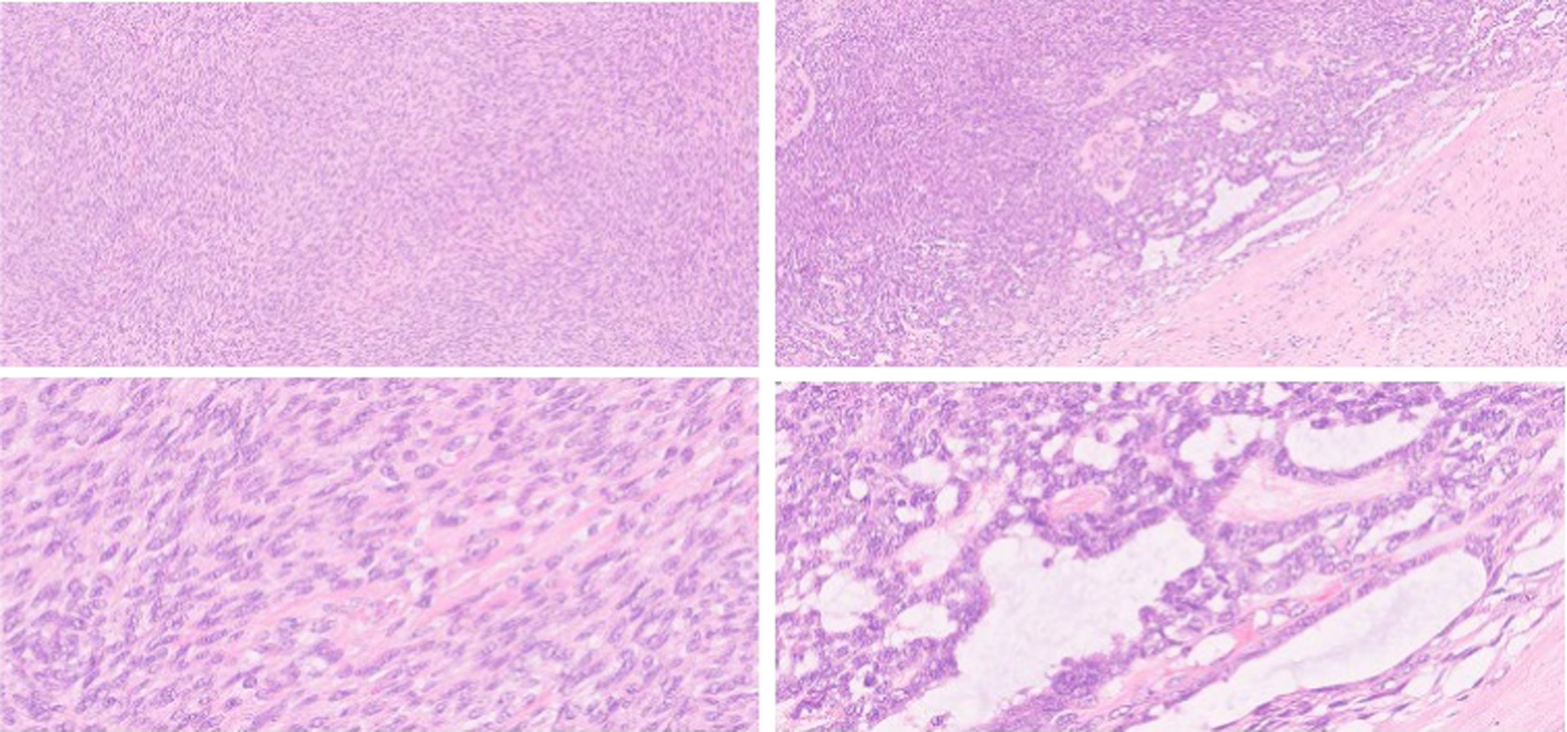
The neoplasm was mostly composed of spindle‐cell areas (upper left image), with glandular‐like structures (upper right image). Areas of hyalinized stroma can be appreciated in both images. No mitoses, atypia or necrosis were seen (H&E, x100 [upper images], ×400 [lower images]).

**FIGURE 4:**
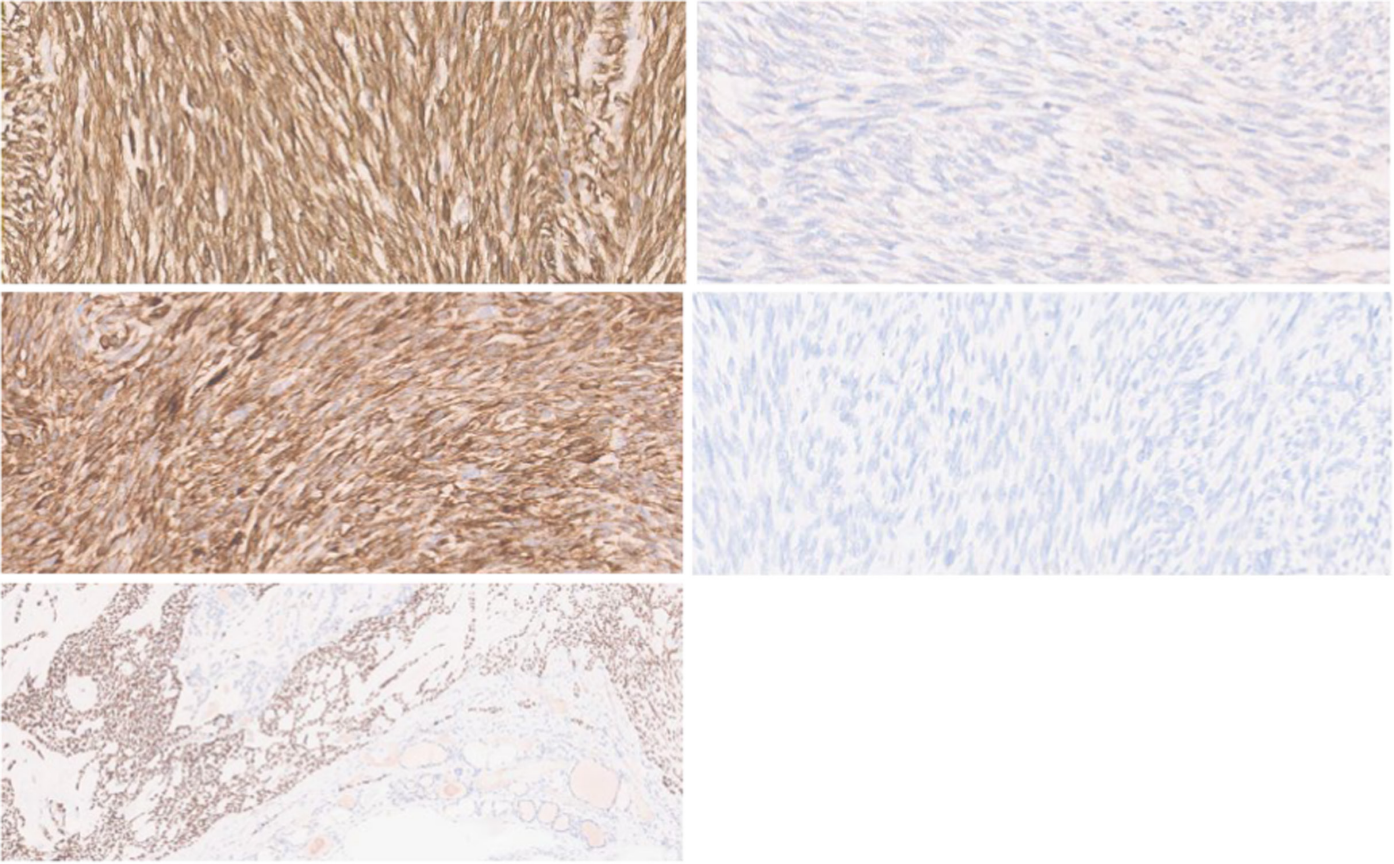
Immunohistochemistry (×400) revealed positivity for cytokeratin AE1/AE3 (upper left), CAM5.2 (middle left) and P63 (lower left). Calcitonin A (upper right) and TTF‐1 (middle right) were negative.

The macroscopic anatomopathological examination identified a solid, brown, well‐defined tumor in the left thyroid lobe, measuring 8 cm. Microscopic examination revealed a predominantly spindle‐cell biphasic neoplasm, with areas of stromal hyalinization (Figure 3). The spindle cells had scant cytoplasm and elongated nuclei with fine chromatin and inconspicuous nucleoli. The glandular component showed microcystic glandular‐like structures. Mitotic figures were rare, and no necrosis was seen. Both components were positive for cytokeratins (AE1/AE3 and CAM5.2), P63, and P40. TTF‐1, calcitonin A, and thyroglobulin were negative (Figure 4). No SS18 gene rearrangements were detected by FISH. A final histopathological diagnosis of SETTLE was rendered. Subsequently, it was decided that no adjuvant treatment was needed. At 6 months of postoperative follow‐up, the patient remained asymptomatic and with no detected disease recurrence by physical and radiologic examination.

## 5 DISCUSSION

SETTLE is a malignant intrathyroidal tumor believed to arise from ectopic thymic tissue or embryonic remnants of branchial pouches.^1^ It typically occurs in children and young adults, the mean age being 15 years, and is clinically indolent.^1^, ^5^, ^6^ Contrary to the present case, there are only few cases reporting tracheal compression at diagnosis.^1^, ^7^, ^8^ Clinically, nodular goiter, differentiated thyroid malignancy, and medullary carcinoma of the thyroid were initially considered.

Diagnostic histological and cytomorphological features of SETTLE include a highly or moderate cellular aspirate with cohesive and single dissociated spindle cells with bland oval nuclei, absence of amyloid, or significant atypia.^1^, ^9^ However, FNAC hyaline material resembling amyloid may lead to misdiagnosis.^10^

In the reported case, cytological examination was suggestive of a medullary thyroid carcinoma. This carcinoma accounts for less than 10% of thyroid cancers, typically presenting between the fourth to sixth decades of life and is characterized by elevated serum calcitonin levels, contrary to the present case.^11^ The recommended treatment for medullary thyroid carcinoma is surgery and the currently accepted approach is to remove the entire gland along with neck and upper chest lymph nodes, if required.^11^

The main histological differential diagnosis includes ectopic cervical thymoma, synovial sarcoma, anaplastic carcinoma, and spindle cell variant of papillary and medullary thyroid carcinoma.^6^, ^10^ Ectopic cervical thymoma is composed of lymphoblasts admixed with the tumor cells, which are absent in SETTLE.^4^, ^9^, ^10^ In synovial sarcoma, the spindle cells are generally more monomorphic, with more mitoses, and immunopositivity for CK is patchy, while in SETTLE CK staining is strong and diffuse.^4^, ^9^ Also, Folpe et al. concluded that molecular genetic detection of synovial sarcoma‐associated fusion genes resulting from the chromosomal translocation t(X;18), can be used for this distinction.^4^ Contrary to SETTLE, anaplastic carcinoma reveals extrathyroidal invasion and extensive pleomorphism, mitoses, and necrosis, and does not usually present in young people.^10^ The striking difference between the medullary or papillary carcinoma and SETTLE is observed in the positive staining of CK and negative staining of typical thyroid gland markers.^4^, ^9^, ^10^ Immunohistochemistry is, therefore, a well‐established tool for differential diagnosis.^7^

Non‐metastatic SETTLE most frequent reported surgical treatment is hemithyroidectomy.^5^ When in doubt, further investigation with frozen section analysis may help to manage the intraoperative strategy.^12^

Despite the low malignant potential, SETTLE has a tendency for late metastasis and the incidence increases significantly as the period of follow‐up overtakes 5 years.^2^, ^13^ To date, the latency between diagnosis and detection of metastasis varies from a few months after the primary tumor manifestation to 25 years.^7^, ^8^ Hence, long‐term follow‐up is recommended to detect possible distant metastasis, in particular to the lungs.^6^, ^7^, ^8^

In conclusion, SETTLE should be considered in the differential diagnosis of thyroid gland malignancies, specially, as in the present case, the patient presents at a younger age, with normal serum calcitonin and FNAC of thyroid nodule suggestive of medullary carcinoma. The clinical implications for treatment, as well as for prognosis, may be significantly different. This case also highlights the importance of histopathological examination in the diagnosis of thyroid cancer. Finally, SETTLE is a rare clinical entity, so future studies are necessary to better understand the pathophysiology, therapeutic choices, and prognosis.

## AUTHOR CONTRIBUTIONS

**Patrícia Bernardo:** Conceptualization; data curation; formal analysis; investigation; methodology; project administration; validation; writing – original draft; writing – review and editing. **Henrique Messias:** Funding acquisition; methodology; supervision; validation; visualization; writing – original draft; writing – review and editing. **Ricardo Nogueira:** Conceptualization; methodology; project administration; supervision; validation; writing – review and editing. **Brazão Lopes:** Data curation; formal analysis; investigation; writing – original draft. **Pedro Gomes:** Project administration; supervision; validation; visualization.

## FUNDING INFORMATION

The authors declare that they have not received any funding for the development of this scientific work.

## CONFLICT OF INTEREST STATEMENT

The authors declare to have no conflicts of interest in connection with this scientific work.

## CONSENT

Written informed consent was obtained from the patient to publish this report in accordance with the journal's patient consent policy.

## Data Availability

The authors declare that all data supporting the findings of this study are available within the article and its supplementary information files.
